# Evolving Concepts in Gestational Iodine Requirements

**DOI:** 10.3390/healthcare14091153

**Published:** 2026-04-25

**Authors:** Charalampos Milionis, Eftychia G. Koukkou, Kostas B. Markou, Ioannis Ilias

**Affiliations:** 1 Department of Endocrinology, Diabetes and Metabolism, Elena Venizelou Hospital, 11521 Athens, Greece; pesscharis@hotmail.com (C.M.); ekoukkou@gmail.com (E.G.K.); 2 Department of Internal Medicine, Division of Endocrinology, University of Patras, 26504 Patras, Greece; markoukonst@upatras.gr; 3 Department of Endocrinology, Hippokration General Hospital, 11527 Athens, Greece

**Keywords:** iodine, pregnancy, public health surveillance

## Abstract

**Highlights:**

**What are the main findings?**

- The maternal thyroid gland can maintain adequate function at urinary iodine concentrations well below the WHO threshold of 150 μg/L, suggesting the current cutoff for pregnant women may be overly conservative.
- Both iodine deficiency and excess follow a U-shaped risk pattern, and functional indices may better capture individual iodine adequacy than traditional biomarkers like UIC and serum thyroglobulin alone.

**What are the implications of the main findings?**

- International guidelines for iodine sufficiency in pregnancy should be revised toward a more physiologically grounded range (closer to 100–199 μg/L), complemented by functional indices rather than relying solely on population-level UIC thresholds.
- Iodine supplementation strategies during pregnancy should shift from universal blanket recommendations toward individualized approaches that account for dietary intake, autoimmune thyroid disease, levothyroxine use, and environmental exposures.

**Abstract:**

Iodine is an essential trace element for thyroid hormone synthesis, metabolic homeostasis, and fetal neurodevelopment. During pregnancy, maternal iodine requirements increase substantially, yet global recommendations are primarily based on population-level biomarkers rather than individualized physiological data. In this review, we examine current international guidelines for iodine adequacy in pregnancy, evaluate the limitations of population-based metrics—such as urinary iodine concentration (UIC) and serum thyroglobulin (Tg)—and highlight emerging evidence on physiological adaptations, functional biomarkers, and individualized risk factors. We incorporated data from population surveillance studies, mechanistic investigations of thyroid adaptation, and clinical outcome research identified through a literature search of PubMed/MEDLINE and Scopus (2016–2025). Evidence indicates that the widely adopted WHO range for iodine intake in pregnant women may overestimate the actual needs of gestation. There is a U-shaped relationship between iodine intake and thyroid outcomes, meaning both low and high iodine exposure adversely affect maternal thyroid function and fetal neurodevelopment, highlighting the narrow optimal intake window. Individualized considerations—including autoimmune thyroid disease, supplementation practices, environmental exposures, and coexisting micronutrient deficiencies—further modulate iodine requirements. Functional indices, such as the Thyroid Feedback Quantile-based Index (TFQI), may offer complementary tools for assessing iodine adequacy beyond traditional biomarkers.

## 1. Introduction

Iodine nutrition during pregnancy remains a critical determinant of maternal thyroid function and fetal neurodevelopment, yet substantial uncertainty persists regarding the optimal criteria used to define iodine adequacy in this population. Current international recommendations are largely based on population-level indicators and extrapolated physiological assumptions, which may not fully capture the dynamic and individualized iodine requirements of gestation. A reassessment of the biological, clinical, and public health frameworks underpinning existing guidelines is therefore warranted.

### 1.1. Physiological Foundations of Iodine

Iodine is an essential trace element with a central role in physiology. It is a fundamental substrate for the synthesis of thyroid hormones (THs)—namely thyroxine (T4) and triiodothyronine (T3)—which regulate protein synthesis, enzymatic activity, metabolic balance, energy homeostasis, and cellular function [1]. The availability of THs is pivotal for optimal neurodevelopment in fetuses and neonates [2,3,4,5]. Thyroid function is tightly regulated by thyroid-stimulating hormone/thyrotropin (TSH), released from the anterior pituitary in response to circulating TH levels. In the setting of insufficient iodine intake, impaired TH synthesis leads to sustained TSH elevation, promoting thyroid hypertrophy and goiter formation [6]. Iodine may also exert extrathyroidal effects, including antioxidant, anti-inflammatory, and immunomodulatory actions [7].

Within the thyroid, iodine is absorbed via the gastrointestinal tract, transported to thyroid follicular cells by the sodium–iodide symporter (NIS), and oxidized in an organification reaction catalyzed by thyroid peroxidase (TPO). Activated iodine is incorporated into tyrosyl residues on thyroglobulin (Tg), generating monoiodotyrosine (MIT) and diiodotyrosine (DIT). TPO-mediated coupling then produces T3 or T4 for secretion into the circulation [8].

### 1.2. Public Health Context and Pregnancy-Specific Considerations

International public health agencies, most prominently the World Health Organization (WHO), have established standardized approaches to assess iodine nutrition at the population level. The WHO endorses urinary iodine concentration (UIC) in spot urine samples as the principal biomarker of population iodine status, reflecting that over 90% of dietary iodine is excreted renally within 24 h. For non-pregnant adults, an appropriate median UIC range of 100–199 µg/L is recommended. Dietary iodine is present in foods of animal and plant origin, marine foodstuffs, iodized salt, and iodine-containing supplements [9,10,11]. The physiological adaptations of pregnancy—including higher demand for TH, renal iodine clearance, and transplacental transfer—substantially increase maternal iodine needs [12]. Iodine deficiency during gestation is linked to adverse neurodevelopmental outcomes in children [13], and iodine intake is often suboptimal among pregnant women globally [14,15].

Despite the well-recognized increase in iodine requirements during pregnancy, current recommendations are largely derived from extrapolation of population-level biomarkers and theoretical assumptions rather than high-quality empirical evidence [16]. In this context, the present article adopts a critical narrative approach to examine existing standards for iodine adequacy during gestation, evaluate the clinical debate surrounding accepted thresholds, and identify the need for updated, physiologically informed international guidelines.

### 1.3. Literature Search

A literature search was conducted in PubMed/MEDLINE and Scopus covering publications from January 2016 to November 2025. The following primary keyword/MeSH term combinations were used: “iodine” AND “pregnancy”; “iodine deficiency” AND “gestation”; “urinary iodine concentration” AND “pregnant women”; “thyroid function” AND “pregnancy” AND “iodine”; “TFQI” AND “pregnancy”; “iodine supplementation” AND “pregnancy outcomes”; “thyroglobulin” AND “pregnancy” AND “iodine”; “iodine excess” AND “pregnancy”. Inclusion criteria: English-language original research articles, systematic reviews, meta-analyses, and clinical guidelines. Exclusion criteria: case reports, editorials, letters, and conference abstracts. Narrative reviews were included selectively when addressing mechanistic or guideline content not available from primary data.

## 2. The Established Paradigm: The WHO Criteria and Implementation Challenges

International policy on iodine nutrition has historically been grounded in population-level surveillance designed to prevent severe iodine deficiency disorders. The WHO has played a central role in defining criteria for iodine adequacy and in promoting standardized assessment tools. These recommendations have guided public health interventions—particularly universal salt iodization—and have contributed substantially to the global reduction of overt iodine deficiency. However, the extension of population-based metrics to physiologically distinct groups, such as pregnant women, introduces important conceptual and methodological challenges.

### 2.1. Population Monitoring and the WHO Threshold

The conventional approach to assessing iodine status at the population level relies on median UIC. The WHO currently defines iodine sufficiency in pregnant women by a median UIC of 150–249 µg/L. This threshold reflects the increased physiological demand during gestation, driven by augmented maternal TH production, transplacental iodine transfer, and increased renal iodine clearance. It is important to acknowledge the rationale underlying the WHO threshold. This range was deliberately set above the estimated average requirement as a conservative public health margin, designed to protect the most vulnerable subgroups—including those with very low habitual intake, limited access to iodized salt, or residing in endemic deficiency areas. In the context of population-level surveillance, such conservatism is epidemiologically justifiable and has historically been effective in reducing the global burden of overt iodine deficiency disorders. Our critique is not directed at this public health logic but rather at the uncritical extension of a population-level surveillance metric as a universal physiological requirement for all pregnant women regardless of their individual circumstances. UIC levels are highly variable and mainly reflect recent iodine intake rather than long-term nutritional status [17]. Spot UIC measurements may be suitable for public health surveillance but are limited in their capacity to assess iodine status in individuals. Accurate individual-level estimation may require up to ten repeated spot or 24-h samples [18,19]. These methodological constraints limit the utility of UIC for personal dietary counseling or therapeutic decision-making during pregnancy.

### 2.2. Supplementation and Persistence of Deficiency

Recognizing the elevated demands during gestation, most major medical organizations recommend that pregnant, preconceptional, and lactating women receive a daily supplement containing 150 µg of iodine [20,21,22]. Despite this consensus, insufficient adherence to supplementation remains a major global public health concern.

Evidence from diverse geographical regions demonstrates that iodine deficiency among pregnant women remains common, even where iodized salt programs or supplementation policies exist. In Montenegro, nearly half of pregnant women exhibited iodine deficiency [23]. In Malaysia, iodine deficiency was present among pregnant women despite adequate status in school-aged children [24]. Comparable findings have been reported in Italy [25] and Lebanon [26]. In Latvia, many commercially available prenatal supplements failed to provide recommended amounts of iodine and selenium [27]. In the United States, suboptimal iodine intakes are frequent and substantial variability exists in the labeled versus actual iodine content of multivitamin formulations [28,29,30]. Studies from China indicate that inadequate iodine knowledge contributes to confusion regarding dietary intake [31,32]. Together, this evidence highlights the need for improved counseling and stronger national regulatory frameworks to ensure consistent iodine provision in prenatal products.

## 3. The Spectrum of Iodine Imbalance: Deficiency and Excess Risks

Maintaining iodine intake within a narrow optimal range is critical during pregnancy, as both deficiency and excess are associated with adverse outcomes. A U-shaped relationship exists between iodine intake and thyroid outcomes: insufficient intake compromises TH synthesis and fetal neurodevelopment, while excessive exposure may induce thyroid dysfunction and developmental harm. This U-shaped pattern has been quantitatively defined: intakes below 150 µg/day or above 550 µg/day are not recommended, based on balance study data [33].

### 3.1. Consequences of Iodine Deficiency

Even mild iodine deficiency has clinically relevant consequences. In a large meta-analysis of pregnant women (*n* = 8261), mild deficiency was associated with alterations in thyroid function, including elevated free T3, free T4, and abnormal thyroglobulin antibody (TgAb) profiles [34]. Iodine deficiency has also been linked to adverse pregnancy outcomes: insufficient intake, especially with mildly elevated TSH levels, has been associated with endothelial dysfunction and increased severity of preeclampsia [35,36]. Preeclampsia is characterized by significantly lower free T3 and free T4 concentrations, reinforcing the complex interplay between iodine status, thyroid function, and placental health [37]. Low iodine intake has also been associated with reduced fecundity [38].

The impact of maternal iodine deficiency extends to the fetus. Maternal iodine status influences neonatal TSH concentrations [39]. Adequate iodine intake through supplementation and iodized salt, particularly before conception and during the first trimester, has been associated with improved neonatal outcomes, including higher APGAR scores [40]. Furthermore, iodine supplementation during pregnancy has been shown to improve psychomotor development indices in children from mild-to-moderately deficient regions [41].

### 3.2. Risks of Excessive Iodine

Conversely, iodine overabundance is also a significant concern, particularly in settings with widespread supplementation or fortified foods. Excessive iodine intake has been associated with impaired infantile development [42,43]. Supraphysiological iodine levels are associated with an increased risk of adaptive developmental delay at 18–24 months of age [44]. Prenatal daily iodine intake outside the range 160–220 µg has been linked to higher maternal anhedonia during the postpartum period [45]. Extreme habitual intakes—below 150 µg or above 550 µg per day—should be avoided to minimize physiological disruption [33].

Excessive iodine may also adversely affect maternal and neonatal thyroid function. Undue iodine exposure during pregnancy has been associated with an increased risk of complications, potentially mediated through iodine-induced thyroid dysfunction [46]. Maternal subclinical hyperthyroidism has been linked to an increased risk of preterm delivery, particularly among women with higher urinary iodine concentrations [47]. Medical exposures—including topical iodine-containing antiseptics used during cesarean delivery [48,49,50] and iodinated contrast media [51,52]—can also result in substantial iodine uptake, transiently disrupting iodine status. Such exposures in the preconception period or early gestation may cause temporary maternal hypothyroidism, with possible effects on the fetus.

## 4. The Imperative for Guideline Revision: Challenging the WHO Threshold

A central controversy revolves around whether the widely adopted WHO cutoff of 150 µg/L in median UIC accurately reflects true iodine adequacy during gestation. Recent evidence, particularly from studies examining physiological indicators of thyroid function, suggests this threshold may be more stringent than necessary. Table 1 summarizes current recommendations from major international organizations and compares them with the physiologically reasoned range proposed herein.

### 4.1. Physiological Adaptations and Thyroid Strain

The maternal thyroid axis exhibits a remarkable adaptive capacity that appears to have been underestimated in current guidelines. This is demonstrated by the nationwide Faroese study by Johannesen et al. (2025) [57]. In a cohort of 623 pregnant women (representing 63% of all pregnancies in the country during 2020–2022), the median UIC was 108 µg/L—below the WHO threshold—yet clinically significant thyroid strain, indicated by elevated serum Tg, was observed only when UIC fell below 50 µg/L. TSH was not elevated in any UIC stratum. The study had a robust nationwide cross-sectional design; however, its limitations include the single time-point UIC measurement (which has poor individual precision), the seafood-rich Faroese dietary context (which may limit generalizability to other populations), and its cross-sectional design (which precludes causal inference about outcomes). Nevertheless, these results suggest that the thyroid gland can maintain adequate hormone production at lower iodine concentrations than currently recommended, warranting reconsideration of international standards.

### 4.2. Limitations of Traditional Biomarkers

Assessment of iodine status during pregnancy is complicated by the variable reliability of traditional biomarkers. Serum Tg, though commonly used as a long-term indicator of iodine status in the general population, has limited utility during gestation [58]. The interpretation of Tg levels is confounded by the unique hormonal milieu of pregnancy, including the TSH-like activity of human chorionic gonadotropin (hCG), which transiently modulates thyroid function. Elevated serum Tg may also occur for unclear physiological reasons during gestation [59,60].

Furthermore, the rapid hormonal changes throughout pregnancy necessitate trimester-specific reference intervals for TSH and free T4 [61,62,63,64]. Reliance on fixed reference limits—such as those historically recommended by the American Thyroid Association (ATA)—can result in delayed diagnosis or inappropriate therapy for a substantial proportion of pregnant women. Accurate assessment of thyroid function in pregnancy therefore requires dynamic adaptation of clinical thresholds based on local, trimester-specific reference intervals.

### 4.3. The Role of Functional Indices

Researchers have explored functional indices that capture the dynamic adaptation of the hypothalamic–pituitary–thyroid (HPT) axis during gestation, to move beyond the limitations of static biomarkers. The Thyroid Feedback Quantile-based Index (TFQI) is a calculated measure derived from routinely available TSH and free T4 values; it estimates the central (hypothalamic-pituitary) sensitivity to thyroid hormone feedback. In the study by Ilias et al. (2024) [65], 1102 trimester-specific blood and urine samples were collected from pregnant women without known thyroid disease. TFQI correlated negatively with UI in the first trimester (Pearson r: −0.323, *p* = 0.04) and positively for UI values above 250 µg/L in the second trimester (r: +0.368, *p* = 0.050). These results suggest that the HPT axis adapts to iodine intake in a trimester-dependent manner, and that complex HPT axis changes are poorly represented by a single median UIC threshold. The TFQI has important current limitations that must be acknowledged when considering its clinical application. First, validation data derive from relatively small, single-center cohorts; population-level reference ranges have not been established. Second, trimester-specific TFQI reference intervals are not yet standardized across different populations or assay platforms. Third, TFQI has not been evaluated as a clinical decision tool in prospective outcome trials—that is, no study has yet demonstrated that TFQI-guided supplementation improves maternal or fetal outcomes compared with conventional UIC-based management. Fourth, its calculation requires paired TSH and free T4 measurements on the same sample, which, while routine in specialist settings, adds analytical complexity in primary care. For these reasons, TFQI should currently be regarded as a research-grade complementary biomarker rather than a clinical standard. Its most appropriate role at present is as a second-level assessment tool in research or specialized clinical settings when conventional biomarkers yield ambiguous results—for example, when UIC appears adequate but unexplained thyroid function changes are present. Larger prospective studies, and ideally a dedicated validation study, are needed before TFQI can be recommended for routine clinical use alongside UIC and Tg.

Table 2 summarizes the key studies directly informing the proposed 100–199 µg/L range. The evidence is consistent in suggesting that the WHO threshold incorporates a margin beyond what is needed to prevent measurable thyroid strain in euthyroid pregnant women; however, the data derive from observational designs, and prospective randomized confirmation is required before these findings can be translated into guideline revisions.

## 5. Clinical and Environmental Factors in Iodine Management During Pregnancy

Optimal iodine management during pregnancy extends beyond general dietary recommendations and population-level guidelines. Maternal iodine status is influenced not only by intake but also by individual health conditions, supplementation practices, and environmental exposures.

### 5.1. Targeted Supplementation and Autoimmune Thyroiditis

While blanket supplementation is the current recommendation, emerging evidence suggests the need for personalized approaches, particularly in areas where adequacy may already be present through diet. Because both low and high iodine intakes can be associated with poorer neurodevelopment in children, universal supplementation raises concerns about potential negative impact on children born to women who already have sufficient dietary iodine intake [66]. The ongoing PoppiE randomized controlled trial [67]—which is comparing 20 µg/day versus 200 µg/day of iodine in 754 pregnant women with pre-existing sufficient intake (>165 µg/day)—is designed to test this hypothesis directly and will provide high-quality prospective evidence. Personalized supplementation under medical supervision is especially advisable for women with low intake of iodine-rich foods, such as those on vegan diets [68,69].

For women with autoimmune thyroiditis receiving levothyroxine (LT4) therapy during pregnancy, iodine supplementation is still required, but the dosage must be carefully calibrated to the individual’s LT4 regimen, because excessive iodine can exacerbate thyroid dysfunction. In one study, newborns of women taking iodine-containing supplements exhibited higher TSH levels compared to those whose mothers did not take such products [70], highlighting the delicate balance required in this high-risk subgroup.

### 5.2. Environmental and Micronutrient Interactions

Optimal iodine management cannot be considered in isolation from other essential micronutrients and environmental exposures, as these factors interact to influence maternal and fetal thyroid function. Selenium is critical for both maternal TH synthesis and fetal neurodevelopment, acting synergistically with iodine [2]. Selenium deficiency remains prevalent in certain populations, and maternal deficits are directly transferred to the newborn, potentially compounding the effects of inadequate iodine [71].

Environmental toxicants also play a significant role in thyroid health. Exposure to heavy metals such as mercury can exacerbate thyroid dysfunction, particularly in the context of dietary iodine deficiency [27,72]. Exposure to non-persistent endocrine-disrupting chemicals, including phthalates and parabens, during pregnancy has been shown to alter neonatal thyroid function [73,74]. These findings reinforce the importance of integrating nutritional, medical, and environmental considerations into individualized iodine management strategies for pregnant women.

## 6. Conclusions

**Figure 1:**
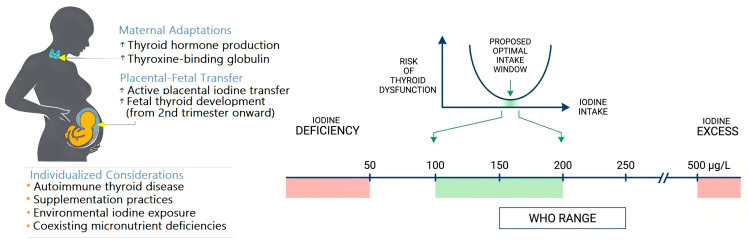
Iodine requirements and thyroid adaptation in pregnancy. Iodine is an essential trace element required for thyroid hormone synthesis. During pregnancy, maternal iodine requirements increase due to enhanced thyroid hormone production and active transplacental iodine transfer to the fetus. A U-shaped relationship exists between iodine intake and thyroid outcomes, whereby both insufficient and excessive exposure are associated with adverse maternal thyroid effects, indicating a narrow optimal intake window. Individual features—including autoimmune thyroid disease, supplementation practices, environmental iodine exposure, and coexisting micronutrient deficiencies—further modify iodine requirements and thyroidal response during gestation. Evidence indicates that the widely adopted WHO range for iodine intake in pregnant women may overestimate the actual needs of pregnant women.

The current WHO criteria for iodine sufficiency in pregnant women, defined by a median UIC of 150–249 µg/L, are difficult to justify on purely physiological grounds. Accumulating evidence—particularly the Faroese nationwide data—indicates that the maternal thyroid gland maintains adequate function at urinary iodine concentrations well below this threshold, with thyroid strain evident only below 50 µg/L. Furthermore, the U-shaped relationship between iodine intake and adverse outcomes emphasizes the importance of avoiding both deficiency and excess, rather than simply maximizing intake to meet population-based thresholds (Figure 1).

**Figure 2:**
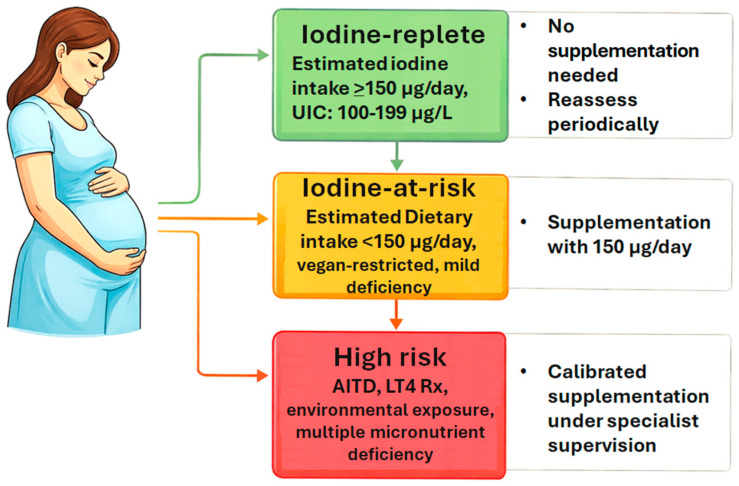
Clinical Risk-Stratification Framework for Iodine Management in Pregnancy; UIC: urinary iodine concentration; AITD: autoimmune thyroid disease; LT4Rx: levothyroxine therapy.

Based on the evidence reviewed, a simplified risk-stratification framework for iodine management in pregnancy can be proposed, as illustrated in Figure 2. Three groups can be delineated. In the first group—iodine-replete women (estimated dietary intake ≥150 µg/day or UIC 100–200 µg/L, no specific risk factors, residing in an iodine-sufficient region)—universal supplementation may not be necessary, and reassessment of dietary intake with monitoring is preferred. In the second group—women at risk of iodine insufficiency (dietary intake <150 µg/day, vegan or severely restricted diet, residing in a mild-deficiency region)—supplementation with 150 µg/day is recommended. In the third group—high-risk women with individualizing factors (autoimmune thyroid disease on LT4, multiple micronutrient deficiencies, significant environmental exposures, LT4 dose adjustments during pregnancy)—individualized supplementation under specialist supervision is advised, with dose calibration guided by repeated UIC measurements and, where available, functional indices. This framework is intended as practical guidance rather than a formal clinical protocol, pending confirmation from prospective randomized trials such as PoppiE.

We wish to be explicit that the physiologically reasoned 100–199 µg/L range proposed herein represents a hypothesis-generating conclusion based on observational and cross-sectional data, not a guideline-grade recommendation (Figure 1 and Figure 2). Confirmation through prospective randomized trials—such as PoppiE—and larger multi-population studies is required before any threshold revision can be formally recommended. Future international guidelines should incorporate: (1) trimester-specific and laboratory-specific reference intervals for TSH and free T4; (2) individualized supplementation strategies stratified by dietary intake, autoimmune status, environmental exposure, and comorbid conditions; (3) complementary functional biomarkers such as TFQI, once adequately validated; and (4) a recalibrated population surveillance threshold that acknowledges the thyroid’s adaptive capacity during gestation. In the interim, clinicians should approach iodine supplementation in pregnancy with care and caution—maintaining a low threshold for supplementation in at-risk groups, while avoiding unnecessary supplementation in women with documented adequate dietary intake.

## Figures and Tables

**Table 1:** Comparison of current international recommendations on iodine sufficiency in pregnancy with the authors’ proposed range and identified evidence gaps.

Organization	Recommendation	Threshold/Target	Biomarker Used	Evidence Gaps
WHO (2007/2013) [53]	Population iodine sufficiency in pregnancy	Median UIC 150–249 µg/L	Spot UIC (population median)	Based on extrapolation; does not account for HPT axis adaptation or functional indices; no individualized assessment
ATA (2017) [54]	Supplementation 150 µg/day during pregnancy/lactation	Dietary intake ≥250 µg/day total	Dietary intake estimate; UIC	Threshold extrapolated from population data; U-shaped risk not fully addressed
ETA (2014/2022) [55]	Supplement 150–200 µg/day; avoid excess	UIC 150–249 µg/L; avoid >500 µg/L	UIC; Tg	Limited prospective outcome data; TFQI not considered
Endocrine Society [56]	Supplement if dietary intake <250 µg/day	Total intake ≥250 µg/day	Dietary assessment	Individualized factors (autoimmune disease, LT4 therapy) not stratified
Authors’ proposed range	Risk-stratified supplementation; revise UIC threshold	UIC closer to 100–199 µg/L; thyroid strain only below 50 µg/L	UIC + functional indices (TFQI); Tg; dietary assessment	Requires prospective RCT validation; not yet guideline-grade

ATA, American Thyroid Association; ETA, European Thyroid Association; HPT, hypothalamic–pituitary–thyroid; Tg, thyroglobulin; TFQI, Thyroid Feedback Quantile-based Index; UIC, urinary iodine concentration; WHO, World Health Organization.

**Table 2:** Key studies supporting the proposed revision of the WHO UIC threshold in pregnancy.

Study	Design/*n*	Primary Outcome	Main Result	Limitations
Johannesen et al. 2025 [57]—Faroese study	Cross-sectional; *n* = 623 pregnant women (63% of national pregnancies)	Serum Tg and TSH as indicators of thyroid strain	Tg elevated only with UIC < 50 µg/L; no TSH elevation at any UIC level; median UIC 108 µg/L	Single time-point UIC (poor individual precision); seafood-rich diet may limit generalizability; cross-sectional design precludes causal inference
Ilias et al. 2024 [65]—Greek cohort	Prospective observational; *n* = 1102 trimester-specific samples	TFQI-UI correlation across trimesters	TFQI negatively correlated with UI in T1; positively correlated in T2 for UI > 250 µg/L; HPT axis adaptation occurs below 150 µg/L	Sample attrition by trimester; TFQI reference ranges not yet population-standardized; no clinical outcomes assessed
Chen et al. 2023 [33]—Chinese balance study	Metabolic balance study; *n* = 113 pregnant women	Iodine balance across intake levels	Intakes <150 µg/day or >550 µg/day associated with negative or supra-positive iodine balance; optimal 150–550 µg/day range	Short-term study; balance does not equal clinical outcomes; Chinese diet and iodization context may differ from other populations

Tg, thyroglobulin; TFQI, Thyroid Feedback Quantile-based Index; UIC, urinary iodine concentration; HPT, hypothalamic–pituitary–thyroid; T1, first trimester; T2, second trimester.

## Data Availability

No new data were created or analyzed in this study. Data sharing is not applicable to this article.

## Abbreviations

The following abbreviations are used in this manuscript:

ATA	American Thyroid Association
DIT	Diiodotyrosine
hCG	Human chorionic gonadotropin
HPT	Hypothalamic–pituitary–thyroid (axis)
LT4	Levothyroxine
MIT	Monoiodotyrosine
NIS	Sodium–iodide symporter
T3	Triiodothyronine
T4	Thyroxine
Tg	Thyroglobulin
TgAb	Thyroglobulin antibody
TH	Thyroid hormone(s)
TFQI	Thyroid Feedback Quantile-based Index
TPO	Thyroid peroxidase
TSH	Thyroid-stimulating hormone/Thyrotropin
UIC	Urinary iodine concentration
WHO	World Health Organization
